# Improving Cycle Life of Ni‐Rich Li‐Ion Battery Cathodes by Using Compartmentalized Anode and Cathode Electrolytes

**DOI:** 10.1002/smll.202410149

**Published:** 2025-02-14

**Authors:** Jianqi Sun, Bo Wen, Yaogang Li, Hongzhi Wang, Michael De Volder

**Affiliations:** ^1^ Department of Engineering University of Cambridge Cambridge CB3 0FS UK; ^2^ State Key Laboratory for Modification of Chemical Fibers and Polymer Materials College of Materials Science and Engineering Donghua University Shanghai 201620 P. R. China

**Keywords:** cathode electrolyte interphases, hybrid electrolytes, interfacial engineering, lithium batteries, nickel‐rich cathodes

## Abstract

As new, ever more energy‐dense battery materials are being developed, it is becoming increasingly challenging for electrolytes to cater for both requirements set by the anode and cathode. Ethylene carbonate plays a key role in forming a stable solid‐electrolyte‐interphase on the graphite anodes, but in combination with nickel‐rich cathodes, such as LiNi_0.8_Mn_0.1_Co_0.1_O_2_ (NMC811), it leads to excessive cathode oxygen loss at high voltage. In this work, the study proposes a cell design, where different electrolytes are compartmentalized in the anode and the cathode. An ionically conductive polymer electrolyte membrane is used to prevent two electrolytes from mixing. NMC811 versus graphite full cells using this electrolyte achieved a capacity retention of 85.1% over 520 cycles compared to 61.7% for control systems using a standard carbonate electrolyte under the same conditions. In addition, it is shown that cells using the compartmentalized electrolyte show less transition metal cross‐over from the cathode to the anode and less impedance build‐up during cycling. Overall, this cell design proposed in this work allows to independently optimize the anode and cathode electrolyte and holds the promise to better support the diverging electrolyte requirements of next‐generation anodes and cathodes.

## Graphical abstract

As new, ever more energy‐dense anodes and cathodes are being developed, it is becoming tricky to find a single electrolyte formulation that optimally supports the operation of both electrodes. To address this awkwardness, a sort of new cell design is proposed, where different electrolytes are compartmentalized in the anode and the cathode.

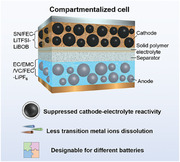

## 1 Introduction

Over the past few decades, Li‐ion batteries (LIBs) have become the preferred technology to power portable electronic devices and electric vehicles.^[^ ^1^ ^]^ The demands for better performance, affordability, and sustainability placed on LIBs by various applications are driving rapid advancements in cathode and anode chemistries.^[^ ^2^, ^3^, ^4^, ^5^ ^]^ So far, electrolyte solvents developed over 30 years ago, which consist of a cyclic (typically ethylene carbonate, EC) and a linear (typically ethyl methyl carbonate EMC, diethyl carbonate DEC, or dimethyl carbonate DMC) carbonate, have been able to achieve satisfactory cell performance and lifetime.^[^ ^6^, ^7^, ^8^ ^]^ However, as the composition of anodes and cathodes evolves, it is becoming increasingly difficult for the same single electrolyte to support both the anode and cathode operation.

LiNi_x_Mn_y_Co_1‐x‐y_O_2_, (NMC) offers higher capacity and lower costs than the classic layered oxide cathode LiCoO_2_, as such, it is widely adopted by electric vehicles targeting long ranges. Over the past decades, the nickel (Ni) content of NMC electrodes has been systematically increased with decreasing cobalt content in order to improve the sustainability of the batteries as well as to increase energy density and reduce cost.^[^ ^5^, ^9^ ^]^ However, once more than 80% of Ni is used (LiNi_0.8_Mn_0.1_Co_0.1_O_2_ or NMC811), these batteries suffer from a substantial decrease in cycling stability or lifespan.^[^ ^10^, ^11^, ^12^ ^]^ Our group and others have previously shown that EC in particular undergoes dehydrogenation reactions coupled with a substantial increase in oxygen loss from the NMC811 surface at high voltage compared to electrolytes using only linear carbonates such as EMC.^[^ ^13^, ^14^, ^15^ ^]^ This leads to a thicker reduced surface layer, continuous increase in cell polarization as well as a cascade of other degradation processes. While doping, optimizing fabrication, modifying, or coating Ni‐rich electrode surfaces can enhance their stability,^[^ ^16^, ^17^, ^18^, ^19^, ^20^, ^21^, ^22^ ^]^ these approaches pose challenges of their own, and ideally these approaches would be used in combination with EC‐free electrolyte formulations. Unfortunately, EC plays an important role in the graphite anode solid‐electrolyte‐interphase (SEI) formation and stabilization, and therefore cannot be simply left out.^[^ ^23^, ^24^ ^]^

In this work, we propose to use an ionically conductive polymer membrane to separate the anode and cathode electrolyte and use standard carbonate electrolyte with EC and additives for graphite on the anode side, and a nitrile‐based electrolyte on the cathode side. Nitriles have been used as electrolyte additives to enhance electrode stability,^[^ ^25^, ^26^, ^27^ ^]^ but they are usually solid or too viscous at room temperature for the simple use. However, they can be mixed with salts to form deep eutectic electrolytes that offer excellent high‐voltage stability, making them a promising candidate to be compatible with nextgeneration cathode materials.^[^ ^28^, ^29^, ^30^ ^]^ In our optimized cathode electrolyte, we use a dual salt eutectic succinonitrile (SN) based system. Previous work suggests that due to the presence of a lone pair of electrons on the N atom in the carbon‐nitrogen bond, it effectively assists in the dissociation of lithium salts, thereby enhancing the Li ions transference number in the electrolyte.^[^ ^31^, ^32^, ^33^ ^]^ Despite these advantages, nitriles normally exhibit poor compatibility with the anodes due to their propensity to undergo nitrile‐based polymerization reactions on lithium metal or graphite anodes, which leads to excessive cell polarization and overall worse cycling stability.^[^ ^34^, ^35^ ^]^

**Figure 1:**
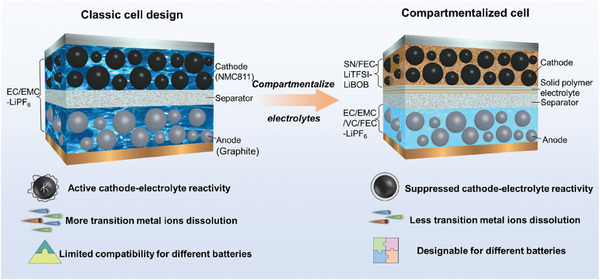
Schematic illustration for the configuration of LIBs with single electrolytes (left) and compartmentalized hybrid electrolyte (right).

The use of different anode and cathode electrolytes has been demonstrated in hybrid solid‐state electrolytes, which face challenges like thickness and speedy ionic conduction of their own.^[^ ^36^, ^37^, ^38^, ^39^ ^]^ To our knowledge, this concept has not been implemented with liquid electrolytes due to challenges in preventing the electrolytes from mixing. We solve this issue by using a thin polyvinylidene fluoride (PVDF) based solid polymer electrolyte (SPE) membrane that separates the two electrode sides combined with controlling the electrolyte amount as illustrated in **Figure** **1**. While this additional membrane complicates the cell assembly, it increased the capacity retention of our cells to 85% over 500 cycles compared to 61.7% when using a commercial carbonate electrolyte under identical conditions. We also observed a suppression of dissolved transition metal (TM) cross‐over (Figure S1, Supporting Information) and less impedance build‐up during cycling.

## 2 Results and Discussion

**Figure 2:**
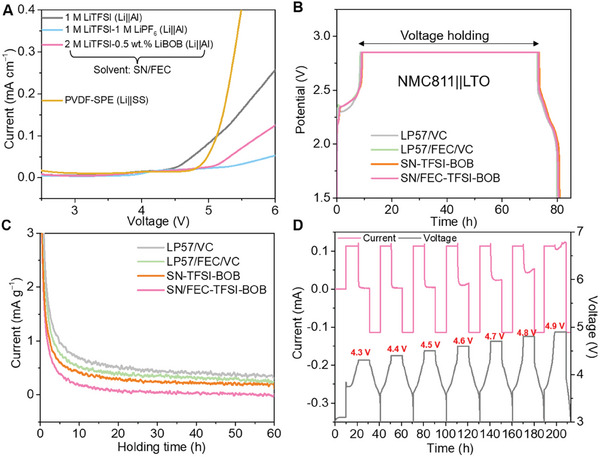
A) Electrochemical oxidation limit of SEEs against aluminum current collector together with the linear sweeping voltammetry curves of solid polymer electrolyte. B) Representative voltage profiles of the voltage holding tests of NMC811||LTO batteries using different electrolytes. C) Normalized currents comparison with different electrolytes during the voltage holding. D) Electrochemical floating analysis of the SEE using the NMC811 cathodes.

Following the above strategy, this work starts by optimizing a SN‐based cathode electrolyte because of its high oxidative stability. We formulate a deep‐eutectic SNbased electrolyte using lithium bis(trifluoromethanesulfonyl)imide (LiTFSI) salt. The salt was selected because it is challenging to achieve high LiPF_6_ concentrations in SN, which brings benefits of higher ionic conductivity. In the LiTFSI‐SN system, better stability in the presence of moisture and high temperature is also seen.^[^ ^8^, ^40^ ^]^ However, LiTFSI is known to cause aluminium (Al) current collector corrosion, and therefore, we start this project by linear sweep voltammetry (LSV) studies of our proposed SN‐based eutectic electrolyte (SEE), with different salt compositions (**Figure** **2**A). For 1 m LiTFSI, the onset of corrosion current starts at 4.3 V, which we anticipate is a result of well‐established corrosion mechanisms between LiTFSI and Al at high voltages.^[^ ^41^ ^]^ Lithium hexafluorophosphate (LiPF_6_) is known to passivate Al, hence we added 1 m LiPF_6_ to the 1 m LiTFSI SEE, which shifts the corrosion onset potential over ≈5.0 V. However, it should be noted that we were unable to fully dissolve 1 m LiPF_6_ into this mixture.^[^ ^42^, ^43^, ^44^ ^]^ Alternatively, increasing the concentration of LiTFSI up to 2 m together with addition of 0.5 wt.% lithium bis(oxalato)borate (LiBOB) as an additive, which is known to passivate Al,^[^ ^45^ ^]^ achieved similar increases in onset potential without manifest solubility issues.

Next, we compare the surface reactivity of NMC811 with carbonate and nitrile‐based electrolytes at high voltage by charging the battery to 4.4 V versus Li and comparing the leakage current after the cell equilibrates. In these experiments, NMC811 is paired with a Li_4_Ti_5_O_12_ (LTO) anode because of its flat voltage profiles and stability. Figure 2B shows the potential profiles of these NMC811||LTO batteries during these experiments (0.1 C charging to 2.85 V versus LTO, a 60‐h voltage hold, and a 0.1 C discharge) and Figure 2C shows the corresponding currents during the voltage hold normalized to the mass loading of NMC811. During the initial 20 hours of voltage hold, the current drops rapidly as the cell equilibrates, after this, the remaining current can be used as a measure for reactions such as lattice oxygen loss from the NMC cathode surface.^[^ ^46^ ^]^ As discussed above, EC used in 1 M LiPF_6_ in EC/EMC (LP57) is known to increase oxygen loss,^[^ ^14^, ^47^ ^]^ and as expected, LP57/vinylene carbonate (VC) shows higher leakage current than our EC‐free SEE electrolyte. Interestingly, we observed that adding 5 wt.% of fluoroethylene carbonate (FEC), which is used to form a stable interphase layer, also decreases the leakage current in our experiments. This is in agreement with reports on FEC improving high voltage stability of electrolytes and electrode‐electrolyte interfacial chemistry.^[^ ^48^, ^49^ ^]^ Based on this observation, we decided to add FEC to our SEE electrolytes, which further reduces the average leakage current by ≈90.8% (Figure S2, Supporting Information). Finally, we investigate the behavior of our proposed SEE electrolyte at high voltage by carrying out electrochemical floating tests from 4.3 to 4.9 V with a step size increment of 0.1 V. As shown in Figure 2D, the leakage current is ≈24.0 µA at an upper cut‐off voltage of 4.7 V but then increases rapidly to ≈53.5 µA at 4.8 V, this represents the optimized SEE electrolytes have a decent endurance on high‐voltage application.

Next, we optimized the PVDF SPE membrane which is critical to separate our anode and cathode electrolytes. These membranes are fabricated by casting a PVDF‐Li dual salts mixture in N,N‐dimethylformamide and drying it, which results in continuous ≈28 µm thick films as shown in Figure S3 (Supporting Information). First, we verified that these films are not dissolved in either our carbonate anode electrolyte or nitrile cathode electrolyte solvents by prolonged soaking experiments shown in Figure S4 (Supporting Information), which should not allow for the cross‐talk of different electrolytes. At room temperature, the ionic conductivity of the PVDF SPE is ≈1.17 × 10^−4^ S cm^−1^ (Figure S5, Supporting Information), and its oxidative decomposition voltage exceeds 4.6 V versus Li (Figure 2A). Then, we measure the Li^+^ transference number of our proposed electrolytes by a combination of current‐time curves together with the AC impedance spectra before and after polarization. The Li^+^ transference numbers of a cell using only the reference carbonate electrolyte (LP57/FEC/VC) and the same anode electrolyte combined with the membrane and SEE cathode electrolyte (compartmentalized SEE‐SPE‐LP57/FEC/VC electrolyte, abbreviated as C‐SPLE) are respectively calculated to be 0.40 and 0.57 (Figure S6, Supporting Information). This improvement may origin from: i) the high concentration of Li salt in the SEE and the ability of SN to dissociate salts enhancing the availability of Li^+^ for charge transport in the electrolyte; ii) the PVDF SPE hampering the movement of anions, which increases the current carried by Li^+^ and hence the transference number. The results above confirm that the C‐SPLE has the potential to increase the battery lifetime without compromising rate performance or cell polarization.

**Figure 3:**
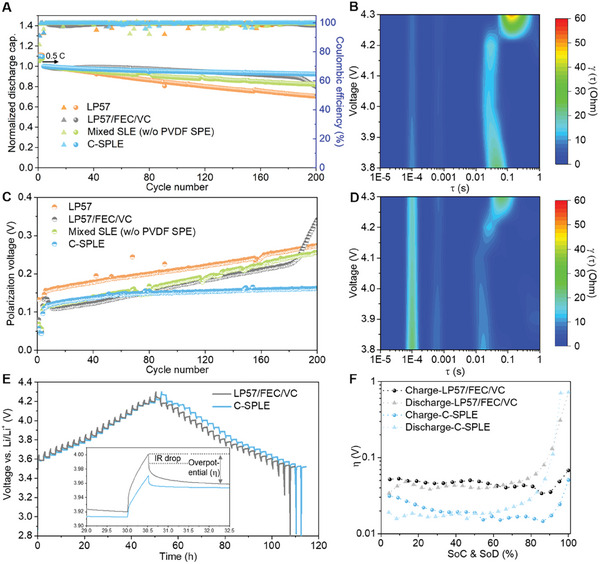
A) Cycling performance for NMC811||Li batteries assembled with different electrolytes at rate of 0.5 C. B) Contour plot of distribution of relaxation times (DRT) based on the impedance spectra at different states of charge using LP57/FEC/VC electrolyte. C) Corresponding voltage polarization of NMC811||Li batteries using different electrolytes. D) Contour plot of DRT based on the impedance spectra at different states of charge using C‐SPLE. E) Voltage profiles of galvanostatic intermittent titration technique measurements on the batteries with different electrolytes, 0.1 C charge/discharge for 30 min and rest for 2 h, the inset is magnified view of one pulse with relaxation. F) Corresponding overpotentials plot as function of states of charge and discharge.

Next, we investigate the cycling performance of our designed electrolytes in half cells as shown in **Figure** **3**A at a rate of 0.5 C and an upper cut‐off voltage of 4.3 V. Standard NMC811 Li‐metal cells using LP57 electrolyte show a capacity retention of 70.4% after 200 cycles. After adding FEC and VC additives to LP57, there is a significant enhancement in the cycling stability for the first one hundred cycles. However, after ≈185 cycles, a knee‐point in capacity decline is observed, ultimately resulting in a capacity retention of 79.7% after 200 cycles. In addition, the cell polarization (difference in nominal charge and discharge voltage) increases steadily in these cells (see Figure 3C), which may be due to oxygen loss of the NMC811 surface in contact with the EC solvent.

In comparison, when using SPE membrane to compartmentalize the electrolytes, we observe a capacity retention of 92.2% under the same cycling condition. Further, we observed that the cell polarization grows rapidly during the first 20 cycles, possibly due to interphase layers formation (and liquid‐solid electrolyte interface/interphase as well), and then stabilizes, which suggests that the cathode surface reduction is stabilized. The impedance spectra at various states of charge of batteries using single LP57/FEC/VC electrolyte and C‐SPLE after 20 cycles were further recorded and presented by distribution of relaxation times (DRT) to decouple the complexed electrochemical steps by capturing the time characteristics (Figure 3B,D; Figure S7, Supporting Information). Resulted from the higher viscosity and lower ionic conductivity of SEE compared with the LP57/FEC/VC (Figure S8, Supporting Information), the density of the peak located at ≈10^−4^ s, which is due to the electric and magnetic effects from the particle‐particle and particle‐current collector interactions,^[^ ^50^, ^51^, ^52^ ^]^ of cell using C‐SPLE is larger than that with LP57/FEC/VC. Because of the same anode electrolyte, there is no large peak intensity difference at time constant of ≈6.4 × 10^−4^ s, which is derived from the SEI layer.^[^ ^51^, ^52^ ^]^ The cathode‐electrolyte‐interphase (CEI) induced peak with time constant of ≈5.0 × 10^−3^ s is observed in C‐SPLE cell earlier than in LP57/FEC/VC cell (Figure S7C, Supporting Information), which is helpful for the subsequent homogeneous ionic transport.^[^ ^53^, ^54^ ^]^ This early formed CEI indicates that the dissociated anions derived from the catholyte enrich the anions concentration within inner Helmholtz layer, thereby potentially facilitating an anion‐driven CEI.^[^ ^55^ ^]^ At the time constant range from 10^−2^ to 10^0^ normally considered as the charge transfer dominated process,^[^ ^56^, ^57^, ^58^ ^]^ the peak intensity of C‐SPLE cell is lower than that of LP57/FEC/VC cell, signifying the lower kinetic barrier that happens to the carriers’ transport through the electrodes and interphase layers.

Batteries with our optimized compartmentalized electrolytes have an average polarization of only ≈0.16 V at 0.5 C after 200 cycles, whereas this is >≈0.25 V for all reference electrolytes (Figure 3C), indicating the lowest accumulated impedance growth. Finally, to confirm the importance of compartmentalizing the electrolytes, we carried out a control experiment where the SPE membrane is removed and as a consequence, the selected anode and cathode electrolytes can mix. In this case, we also see a linear increase in cell polarization, probably due to increases in oxygen loss from the NMC811 surface in the presence of EC, and the capacity retention is reduced to 82.1% after 200 cycles.

Next, we carry out galvanostatic intermittent titration technique (GITT) measurements on cells aged for 20 cycles at 0.5 C using LP57/FEC/VC and C‐SPLE electrolyte, as shown in Figure 3E, also the corresponding overpotentials as the function of states of charge and discharge are plotted in Figure 3F. It can be observed that both of the IR drops (inset in Figure 3E) and overpotentials of the cathode cycled in C‐SPLE are smaller than the one cycled in the reference electrolyte, again suggesting that there is less internal interfacial impedance build‐up in these batteries, which is in agreement with the polarization curves and the current leakage tests.

**Figure 4:**
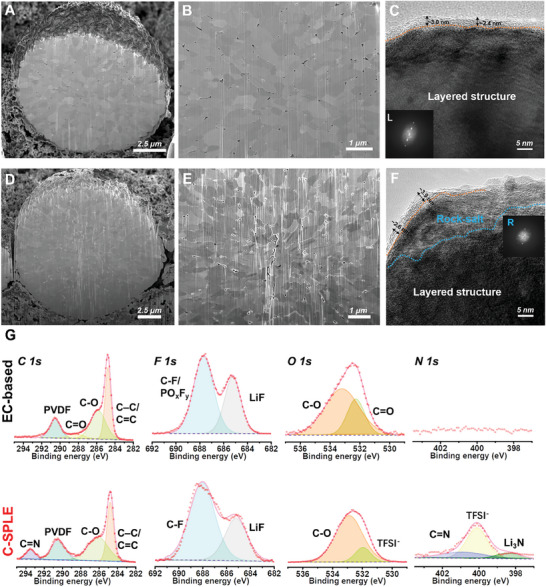
A,B) Focused ion beam (FIB) scanning electron microscopy (SEM) and C) transmission electron microscopy (TEM) images of the cycled NMC811 in C‐SPLE. D,E) FIB‐SEM and F) TEM images of the NMC811 cycled in LP57. G) Typical X‐ray photoelectron spectroscopy spectra for the C 1s, F 1s, O 1s, and N 1s performed on surface of the cycled NMC811 in LP57 and C‐SPLE.

To further clarify the gain effects from C‐SPLE on the cathode physicochemical evolution compared with EC‐containing electrolyte, we study the cathode morphology and surface chemistry using focused ion beam‐scanning electron microscopy (FIB‐SEM), transmission electron microscopy (TEM), and X‐ray photoelectron spectroscopy (XPS), for NMC811||Li batteries aged at 4.4 V for 5 cycles at 0.1 C with different electrolytes. The FIB‐SEM images (**Figure** **4**A,B,D,E) seem to indicate a larger number of intergranular cracks in the cathodes using the reference carbonate electrolyte compared to the C‐SPLE, though a large number of cross sections would need to be made to confirm this. TEM images (Figure 4C and Figure F) indicate a thicker surface reduced layer on the surface and evident rock‐salt phase formation of the NMC811 cycled in LP57, which is in agreement with previous reports on EC‐based electrolytes.^[^ ^46^, ^59^ ^]^ The TEM images also show a surface layer on both samples, which might be the CEI layer, but the one generated within C‐SPLE seems more uniform, this morphology also intuitively illustrates that there is a milder cathode surface reaction with SEE than in the EC‐contained electrolyte (Figure S9, Supporting Information). The above results also prove that, the less oxygen loss and rock‐salt formation resulted from the relatively inert reactivity of the SEE electrolyte against the Ni‐rich cathode can alleviate the boundary shear stress of the primary particles, therefore keeping both of the intergranular and surface integrity, the batteries’ stability will be extended as a further result. In contrast, the cracks due to the electrolyte‐electrode interactions will predictably open up the new reactive surfaces, and then the continuous parasitic reaction will take place on these sites, and finally leading to the growing impedance and sluggish carrier's transport. XPS was used to probe differences in the surface interphases (Figure 4G). For both electrolytes peaks of C─C/C═C, C─O, and LiF can be observed, which are typically attributed to the decomposition of ester solvents, Li salts, and FEC.^[^ ^60^ ^]^ Notably, regarding the F and O 1s spectra of the cathode cycled with the designed catholyte, we find the gradually decreased salt content but the increased LiF with the increase of the etching time (Figure S10, Supporting Information), demonstrating an anion‐driven interphase layer close to the cathode and the salt‐dominated outer layer, this structure may result in the rigid protection for the continuous side‐reaction originated from the cathode (e.g., TM dissolution) but not sacrifice too much ionic conductivity. The largest differences are in the N 1s spectra, where no peaks were observed when using carbonate solvents, the proposed nitrile‐based catholytes show peaks assigned to C═N and possibly Li_3_N.^[^ ^61^ ^]^ By comparison, the suppressed contents of C─C/C═C and C─O species but abundant LiF and emerging Li_3_N tend to form a robust inorganic‐rich interphase layer, which is normally regarded to be more favorable to the overall rapid and uniform ionic diffusion and conduction.

**Figure 5:**
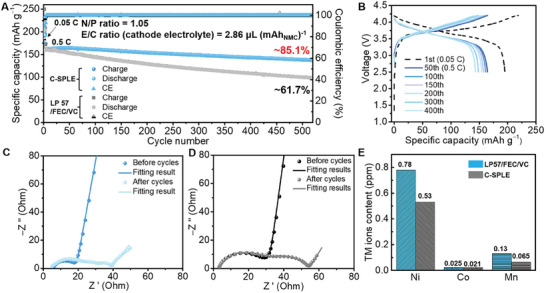
A) Cycling performance for the NMC811||graphite full cells with C‐SPLE and LP57/FEC/VC electrolyte at 0.5 C. B) Typical charge/discharge curves of NMC811/C‐SPLE/graphite full cell at different cycles. AC impedance spectra of the full cells with C) C‐SPLE and D) LP57/FEC/VC before and after 520 cycles. E) Transitional metal dissolution on the cycled graphite anode retrieved from full cells detected by inductively coupled plasma mass spectrometry.

Finally, the advantages offered by C‐SPLE electrolytes are examined in NMC811||graphite full cells. With an N/P ratio of 1.05 and the lean cathode electrolyte condition (E_cathode_/C = 2.86 µL mAh^−1^), the full cells with C‐SPLE can cycle over 520 cycles with a capacity retention of 85.1%, which is ≈23.4% higher than that of the cells using standard LP57/FEC/VC electrolyte (**Figure** **5**A,B). Cells using LP57 without additives or with single additive both degrade faster than C‐SPLE (as shown in Figure S11, Supporting Information). The impedance spectra of full cells before and after cycles were also recorded before and after 520 cycles (Figure 5C,D). Before cycling, the cells with LP57/FEC/VC show a slightly larger total impedance than that of the cells with C‐SPLE, which might be attributed to the higher reactivity between the flooded carbonate electrolyte and electrodes. The impedance spectra reveal that the SPE membrane does not increase the overall cell impedance, this can be further confirmed by the rate performance of full cells (Figure S12, Supporting Information). The C‐SPLE even enables the better performance when cycling rates at 1 and 1.5 C and similar capacity delivery at 2 C, which is related to the higher Li^+^ transference number of C‐SPLE proved above. After the long‐time cycling, the resistance at high frequency especially the charge‐transfer impedance (R_ct_) of the full cells cycled in C‐SPLE are smaller than those of reference cells with LP57/FEC/VC electrolyte, which again suggests that C‐SPLE preventing excessive impedance build‐up. We anticipate that on the cathode, impedance due to oxygen loss from the surface is reduced as demonstrated previously for EC‐free electrolytes,^[^ ^62^ ^]^ as well as possibly the different CEI composition demonstrated by XPS above. On the anode side, both cells are using the same LP57/FEC/VC electrolyte, which is one of slightly optimized standard electrolytes and shows decent compatibilities with various anodes (Figure S13, Supporting Information), and therefore one would expect similar SEI composition and impedance build‐up. However, TM cross‐over is known to catalyze more SEI formation,^[^ ^63^, ^64^ ^]^ and we anticipate that our C‐SPLE electrolyte reduces TM dissolution. The ─C≡N in particular provided by SEE can complex with the TM,^[^ ^65^, ^66^ ^]^ and in addition, the polymer segments of SPE layer may help reducing TM cross‐over. To test this, inductively coupled plasma mass spectrometry (ICP‐MS) was carried out on anodes cycled 520 times with C‐SPLE and reference electrolyte. As shown in Figure 5E, less transition metal ions are deposited when using C‐SPLE. Overall, this work demonstrates that using an SPE membrane has no notable drawbacks but allows for compartmentalizing different electrolytes on the anode and cathodes. This strategy is attractive because new cathode and anode battery chemistries have divergent requirements in electrolyte requirements, such as anode‐free cell systems (Figure S14, Supporting Information). We show that our compartmentalizing strategy allows to suppress battery degradation in NMC811||graphite full cells, leading to batteries with a longer life time and therefore an intrinsic better sustainability.

## 3 Conclusion

In this work, we propose to compartmentalize different electrolytes in the anode and cathode of LIBs for satisfying their diverging requirements. For instance, EC solvent is beneficial to form stable SEIs on graphite anodes, but increases the rate of oxygen loss from Ni‐rich cathodes. In this work, we systematically optimize an EC‐free deep eutectic nitrile‐based electrolyte for NMC811 cathodes and combine it with a classic EC‐containing electrolyte for the anode. In order to prevent those two electrolytes from mixing, we propose using a thin PVDF‐based SPE membrane to compartmentalize them, and we show that this do not lead to additional impedance in the cell. Overall, we achieved a capacity retention of 85.1% over 520 cycles in full cell with our compartmentalized electrolyte system, which is significantly higher than the 61.7% obtained with a standard electrolyte under the same conditions. The development of electrolytes has always been a compromise between anode and cathode requirements and with the development of ever more advanced anodes and cathodes, it is becoming increasingly challenging to strike a balance between the requirements of both electrodes. We believe that the approach to compartmentalize electrolytes proposed in this work provides more freedom in designing electrolytes tailored to the requirements of new anodes and cathodes with ultimately better battery performance and lifetime.

## Conflict of Interest

The authors declare no conflict of interest.

## Supporting information



Supporting Information

## Data Availability

The data that support the findings of this study are available from the corresponding author upon reasonable request.
