# Supplementary material for: Improving Cycle Life of Ni‐Rich Li‐Ion Battery Cathodes by Using Compartmentalized Anode and Cathode Electrolytes
Source: Small. 2025 Feb 14;21(11):2410149. doi: 10.1002/smll.202410149 (PMC11922014; doi:10.1002/smll.202410149)
Supplement: Supplementary file 1 — Supporting Information [file SMLL-21-2410149-s001.docx]

Supplementary information

Improving cycle life of Ni-rich Li-ion battery cathodes by using compartmentalized anode and cathode electrolytes

*Jianqi Sun ^a*^, Bo Wen ^a^, Yaogang Li ^b^, Hongzhi Wang ^b^, Michael De Volder ^a*^*

^a^ Department of Engineering, University of Cambridge, Cambridge CB3 0FS, United Kingdom

^b^ State Key Laboratory for Modification of Chemical Fibers and Polymer Materials, College of Materials Science and Engineering, Donghua University, Shanghai 201620, P. R. China

*Corresponding authors.

Experimental methods

*Electrolytes and electrodes fabrication*

The commercial LP57 (1 M LiPF_6_ in EC/EMC, 3:7 by volume, Targray) and LP57/VC (1 M LiPF_6_ in EC/EMC with 2 wt.% of vinylene carbonate) were used as obtained, 5 wt.% of FEC (Sigma-Aldrich) was added into LP57 or LP57/VC to make reference electrolytes. For the SEE, 0.5 wt.% LiBOB of total mass of SEE was first dissolved into SN (Sigma-Aldrich, 99%, pre-dried at 120^o^C for 2 h before use) at 150^o^C with vigorous stir (LiPF_6_ will be decomposed at this temperature), after cooling to 60^o^C, 2 M LiTFSI (based on the SN, Sigma-Aldrich) was then added and dissolved. 20 wt.% (of total mass with SN, 0.985 g mL^-1^) FEC was added at room temperature at last. For the solid electrolyte membrane, after complexing the same weight of Li salts (LiTFSI:LiDFOB, 7:3 in mass ratio, Sigma-Aldrich) and PVDF (HSV 900, Kynar®) in N,N-dimethylformamide (Sigma-Aldrich, ≥99.9%) solvent at 60^o^C for 72 h, the precursor solution was applied on the aluminium (Al) foil (smooth side) and then spread by doctor blade for twice, the electrolyte membranes was peeled off after drying in vacuum under 60^o^C for 24 h. The SPE membranes should be kept in the glovebox (H_2_O<0.5 ppm, O_2_<0.5 ppm, MBRAUN) at least 24 h before further use.

The cathode electrodes contained 80 wt.% poly-crystalline NMC811 (MTI), 10 wt.% PVDF binder (MTI), and 10 wt.% Super P were coated on the aluminum foil using anhydrous 1-Methyl-2-pyrrolidinone (Sigma-Aldrich) as the solvent, after removing the solvents at 120^o^C, the electrode sheet was punched to 14 mm in diameter and had loadings of ~6.85 and 15.0 (for high-loading batteries only) mg cm^−2^. The slurry contained 87 wt.% LTO (Samsung Fine Chemicals), 5 wt. % Super P, 8 wt.% PVDF binder, and NMP as solvent were coated on aluminum foil, the LTO electrode sheets were punched to 15 mm in diameter after drying the solvent, which had a loading of ~11.1 mg cm^−2^. The graphite anodes water-based slurry contained graphite, sodium carboxymethylcellulose, styrene butadiene rubber, and Super P with a mass ratio of 92:3:3:2 was applied on the copper foil, then the solvent was removed at 120^o^C. The final electrode was punched to 15 mm in diameter, the loading of graphite anode was controlled of ~4.0 mg cm^−2^, which represents a N/P ratio of ~1.05 based on 200 mAh g^−1^ for NMC811 and 360 mAh g^−1^ for graphite.

*Batteries assembly and electrochemical tests*

The linear sweeping voltammetry tests were conducted to investigate the corrosion of different electrolytes against Al and the oxidation limits of solid electrolyte, which is based on the coin cell configuration (CR2032 type, Cambridge Energy Solution) of Al as the working electrode and Li metal as the counter and reference electrodes, the cutoff voltage was set up to 6 V and the sweeping speeds were 1 and 10 mV s^−1^ respectively. The GF/A glass fiber separators (Whatman, pre-dried at 120^o^C overnight before use) were used to make sure the full wettability of various liquid electrolytes, the same separator was used in all liquid electrolytes-based battery tests, unless otherwise specified. The electrolytes (loaded in separator or freestanding SPE) were sandwiched in two stainless steel electrodes to collect the impedance spectra, and then the ionic conductivity of electrolytes was calculated based on the bulk resistance (R_b_), thickness (T) and the effective contact area (A), σ=T/R_b_A. The Li||Li symmetrical cells with C-SPLE and LP57/FEC/VC were assembled, and the chronoamperometry method (oscillation voltage ∆V of 10 mV, aged for 3 h to get the initial I_i_ and steady current I_ss_) together with the recorded charge transfer impedance before (R_i_) and after (R_ss_) polarization were employed to determine the Li^+^ transference number, according to the equation t_Li_^+^=I_ss_(∆V-I_i_R_i_))/I_i_(∆V-I_ss_R_ss_). The coin cells with NMC811 and LTO electrodes and different electrolyte were assembled to conduct the voltage holding tests. The cells were all charged to 2.85 V (in view of the potential of the LTO intercalation plateau is at 1.55 V vs Li/Li^+^) and kept at the same voltage for 60 h, and then discharge to 1.25 V. The CR2032 type coin cells with NMC811 and Li metal foil were used to carry out the voltage floating test for SEE, the upper cutoff voltages were set from 4.3 V with increasing interval of 0.1 V. All the tests above were conducted by VMP3 potentiostat (Biologic).

The NMC811||Li (250 μm) coin cells were assembled to investigate the cycling performances with different electrolytes, distinguishingly for the C-SPLE, 6 μL SEE was first dripped on the cathode, then one piece of SPE membrane was placed on the top of it, and after stacking a layer of glass fiber separators on SPE, 35 μL LP57/FEC/VC was loaded as the anode electrolyte. For the full cell with a graphite anode, as an additional step, 2 μL anode electrolyte was preloaded additionally on graphite anode for filling in the pores inside. The Li metal batteries and full cells were all aged at a rate of 0.5 C after three activation cycles at 0.1 and 0.05 C with cutoff voltage of 2.8-4.3 or 4.4 V and 2.5-4.2 respectively. The LAND cycler was used for the long-term batteries cycling. GITT and impedance spectra of the cycled Li metal batteries at different SoC were recorded after 20 cycles activation. Electrochemical tests and batteries cycling were all carried out in a 25°C climate chamber.

*Materials characterization*

The morphology of the SPE was observed by the by field emission scanning electron microscopy (FE-SEM, Hitachi S-3000N). The intragranular morphology of the aged NMC811 electrodes was observed via FIB-SEM (ZEISS Crossbeam 540), and the further high-resolution TEM images were obtained by Talos F200S. X-ray Photoelectron Spectroscopy was used to study the surface chemical components of the cycled cathodes by Escalab 250Xi. The elemental analysis of the cycled graphite anodes was performed using inductively coupled optical emission plasma spectroscopy (ICP-OES; Thermoscientific) calibrated with standards prepared from an ICP multi-element solution.


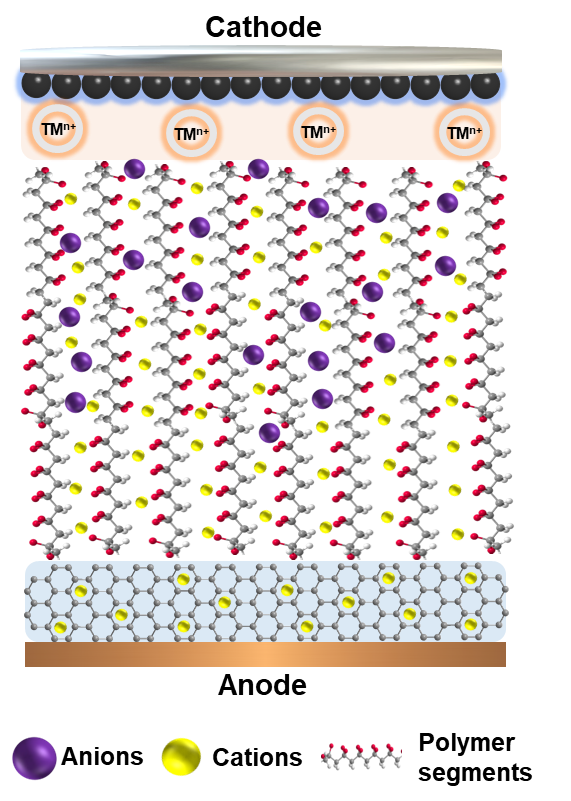


**Figure S1.** Schematic illustration of the proposed suppression effect for TM ions due to the PVDF SPE, the polymeric segment may hinder the rapid crossover of TM ions since their much larger size compared with cations and anions.


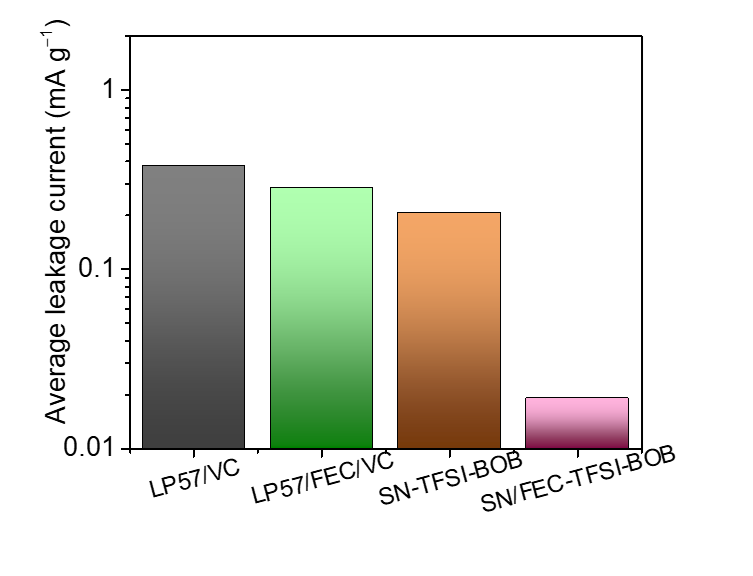


**Figure S2.** Average leakage current during the final 20 h of the voltage holding tests with different electrolytes.

**
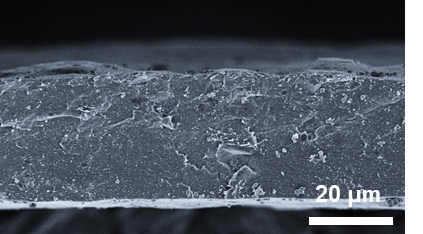
**

**Figure S3.** Cross sectional SEM image of PVDF SPE.


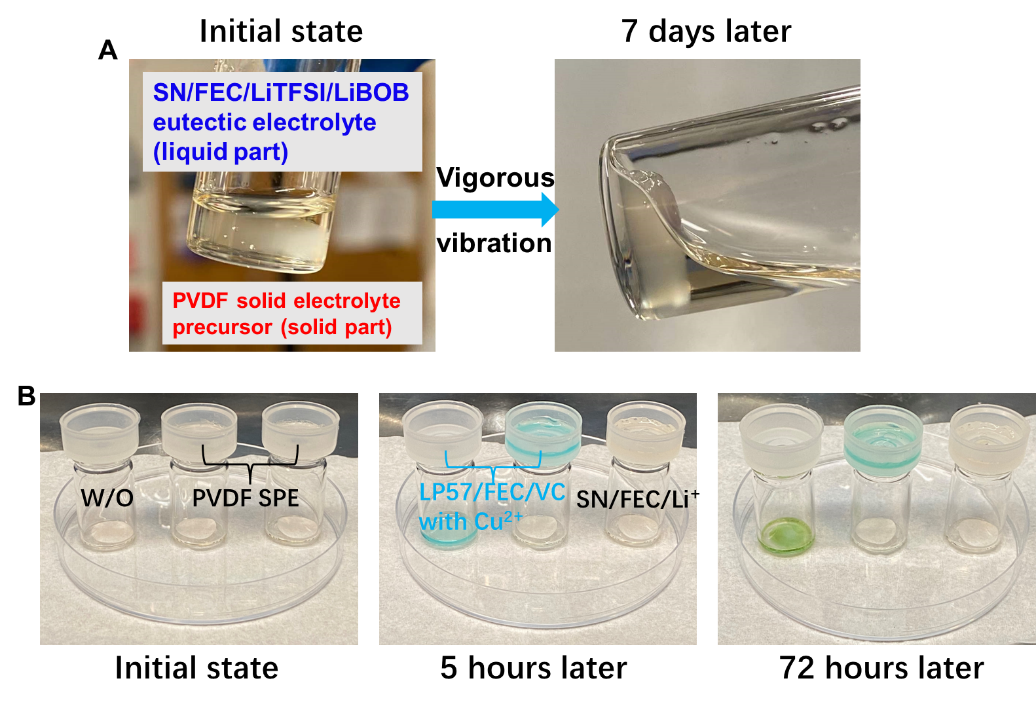


**Figure S4.** Immiscibility tests of PVDF SPE in liquid electrolytes.


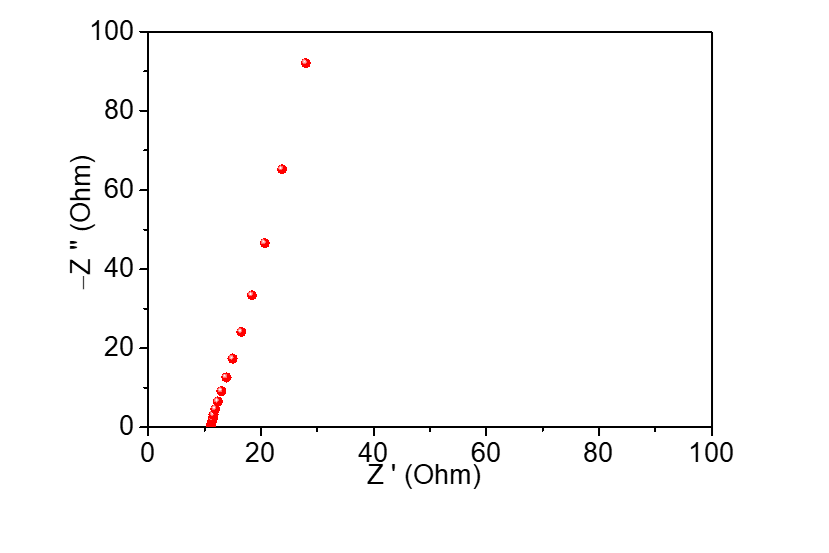


**Figure S5.** AC impedance spectroscopy of the PVDF-based SPE


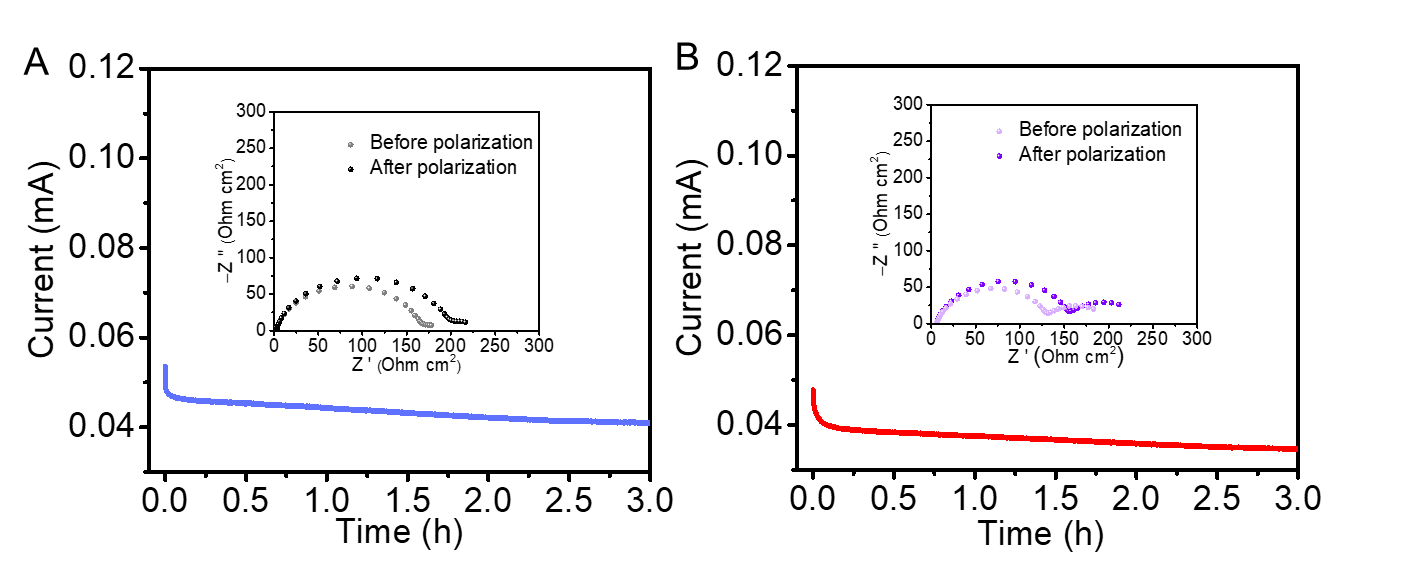


**Figure S6.** Current-time curves following DC polarization of the (A) LP57/FEC/VC and (B) C-SPLE at 10 mV (inset: AC impedance spectra before and after polarization).


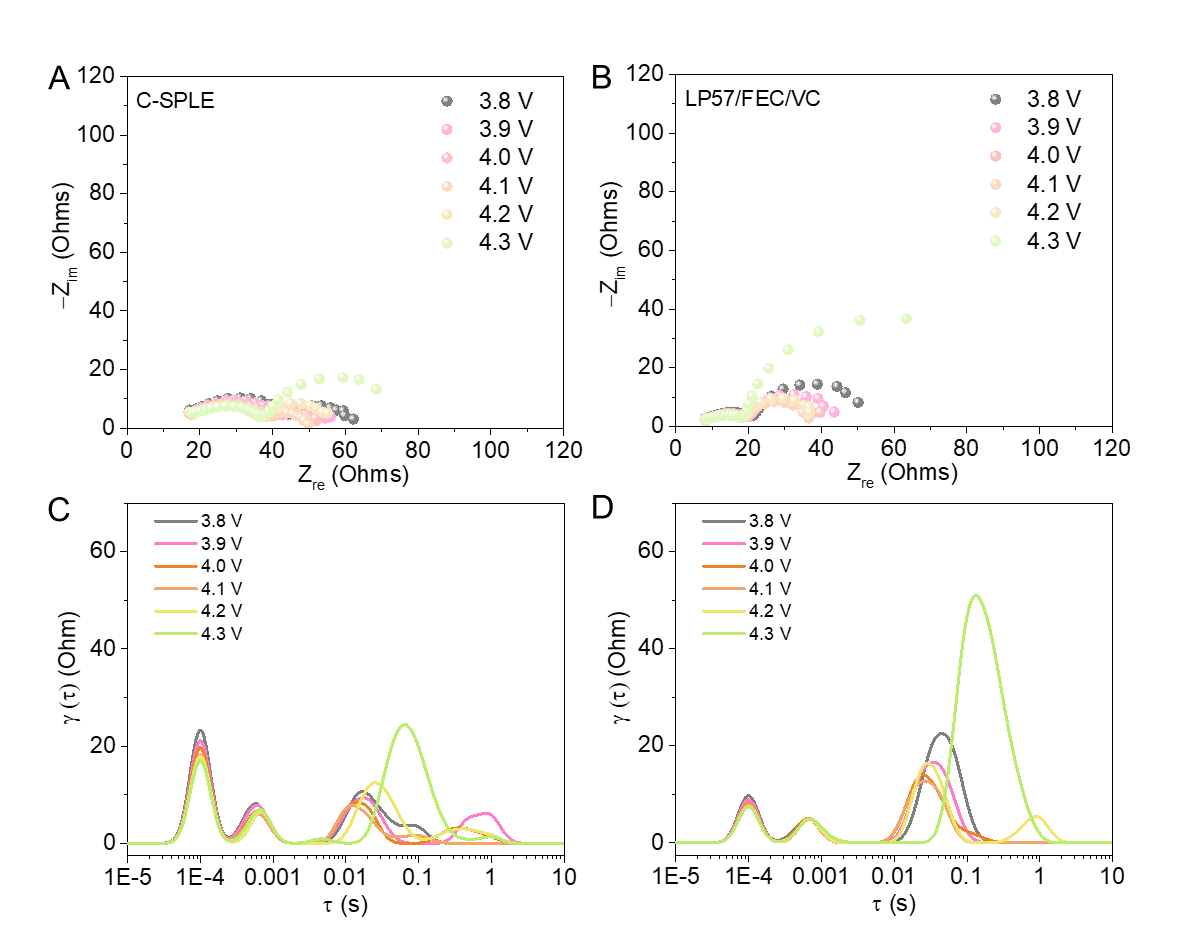


**Figure S7.** Impedance spectra and corresponding DRT plots of batteries cycled with (A, C) C-SPLE and (B, D) LP57/FEC/VC recorded at different states of charge.


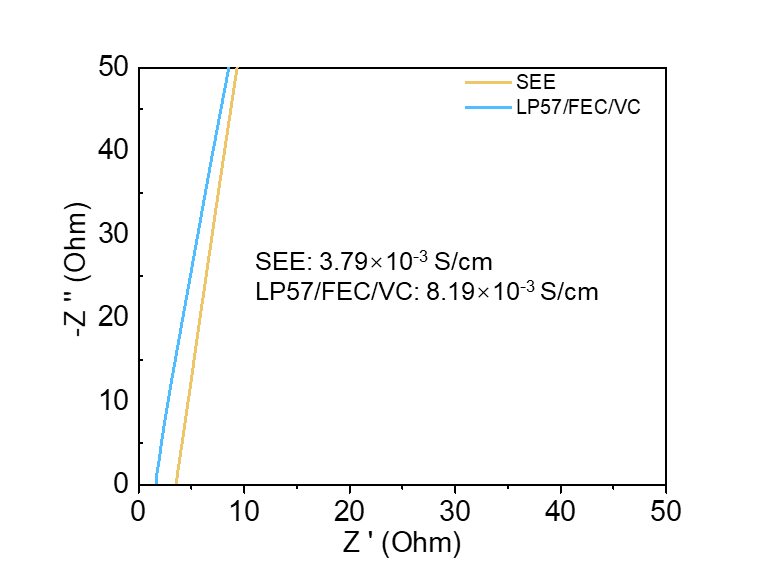


**Figure S8.** AC Impedance spectra of SEE and LP57/FEC/VC.


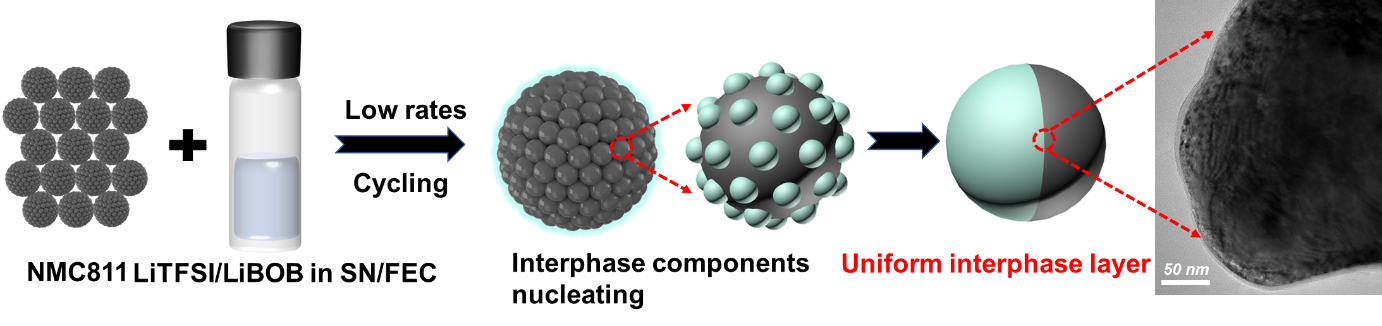


**Figure S9.** Schematic illustration of the interphase layer formation process of the NMC811 cycled with C-SPLE.


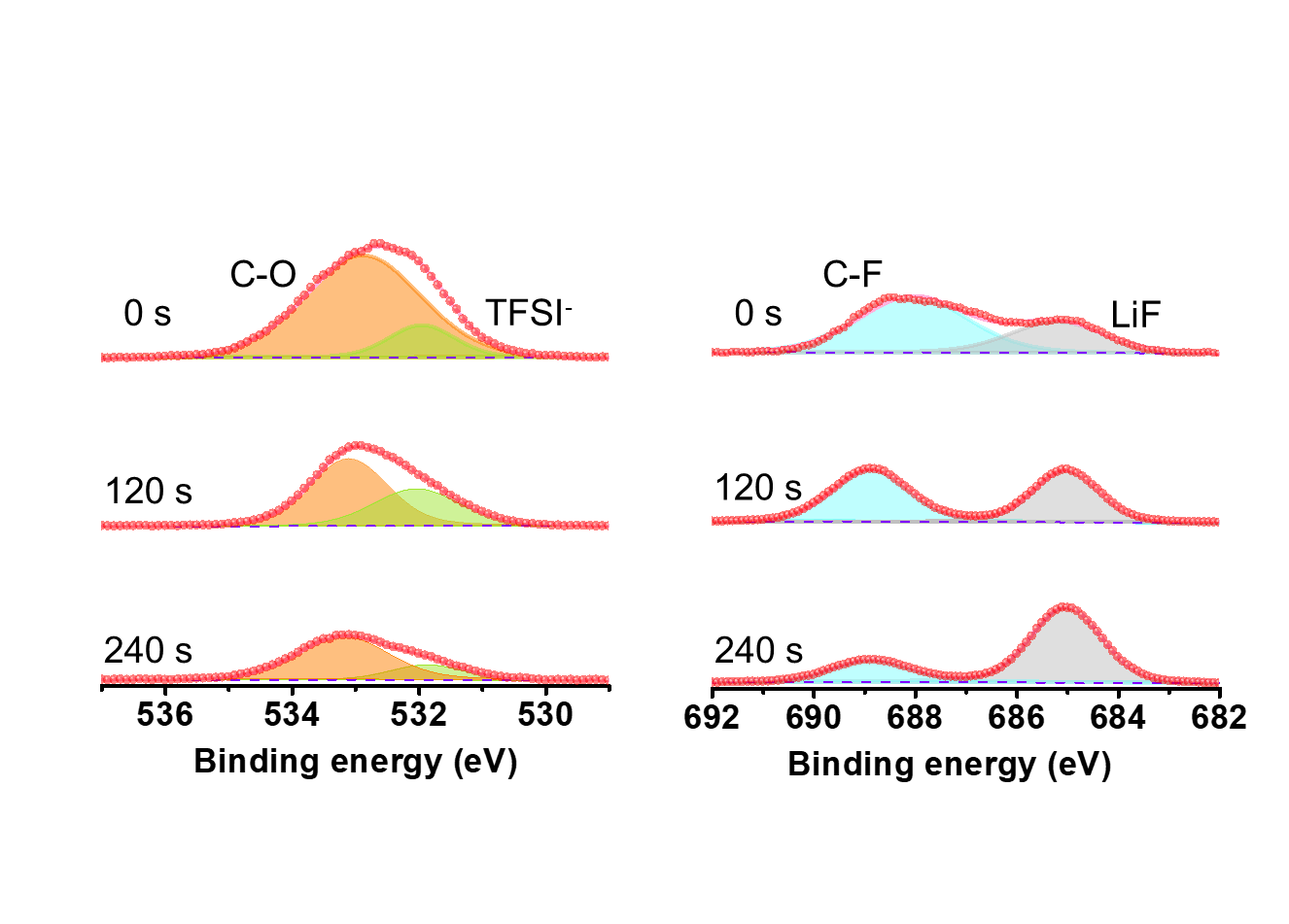


**Figure S10.** XPS characterization with different etching time of the surface on cycled NMC811 using C-SPLE.


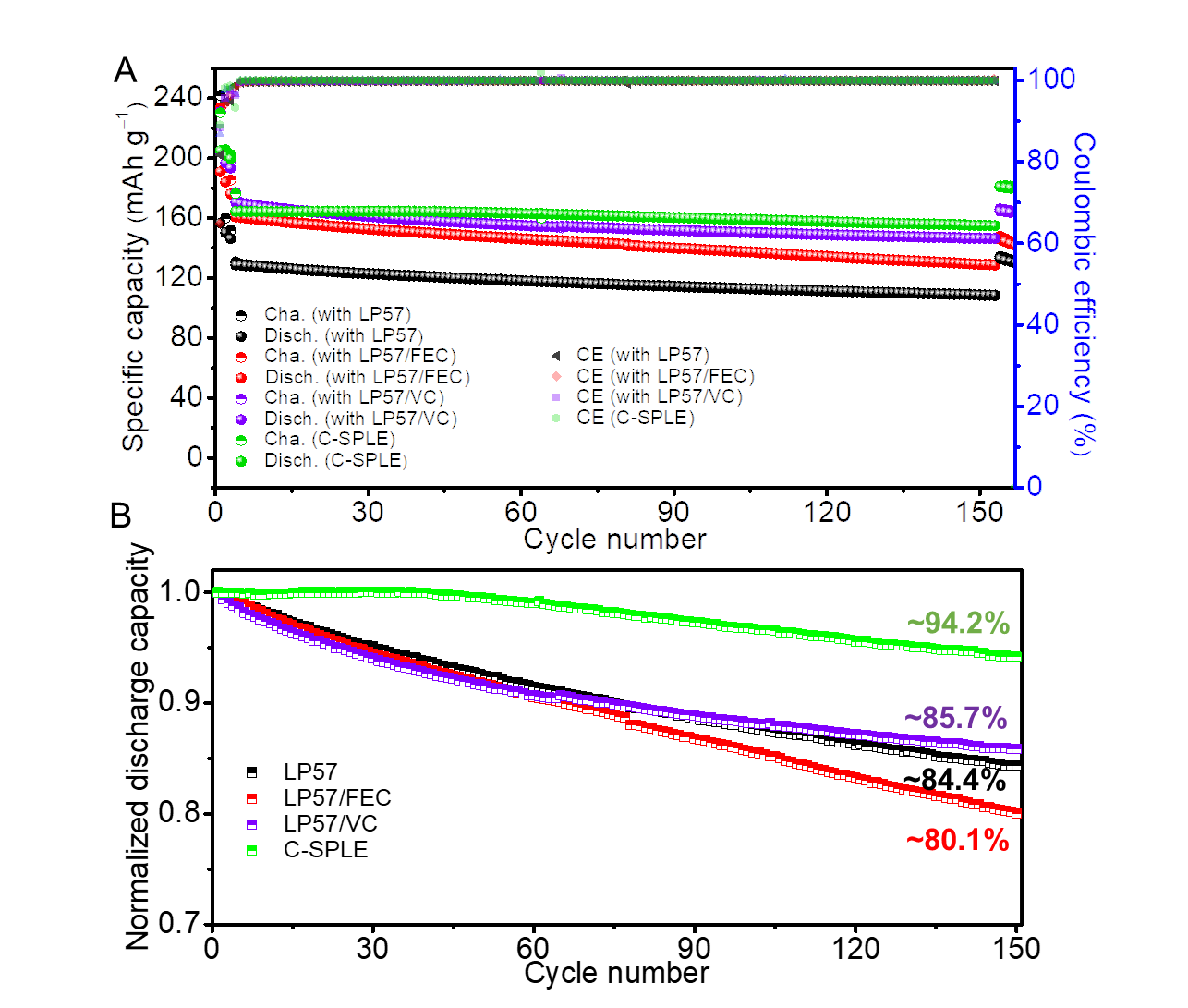


**Figure S11.** (A) Cycling performances of NMC811||Gr batteries with various electrolytes. (B) Normalized capacity retention curves based on the cycling performances.


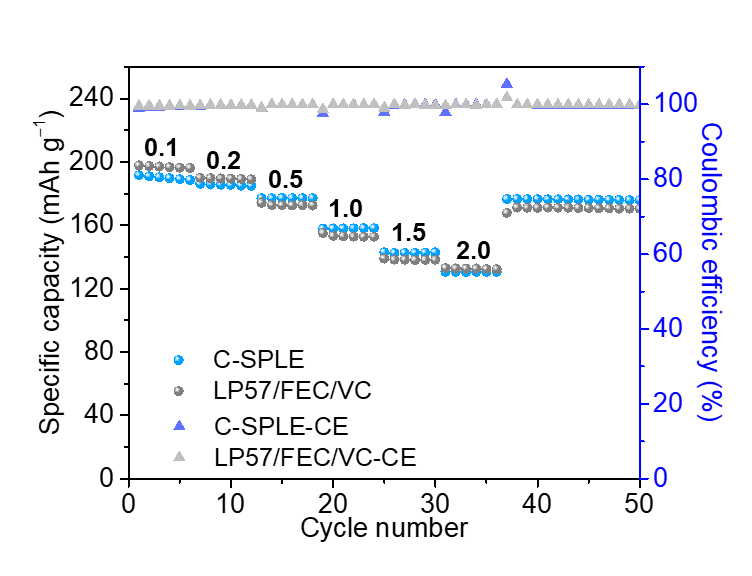


**Figure S12.** Rate performance of NMC811||Gr batteries with C-SPLE and LP57/FEC/VC electrolytes.


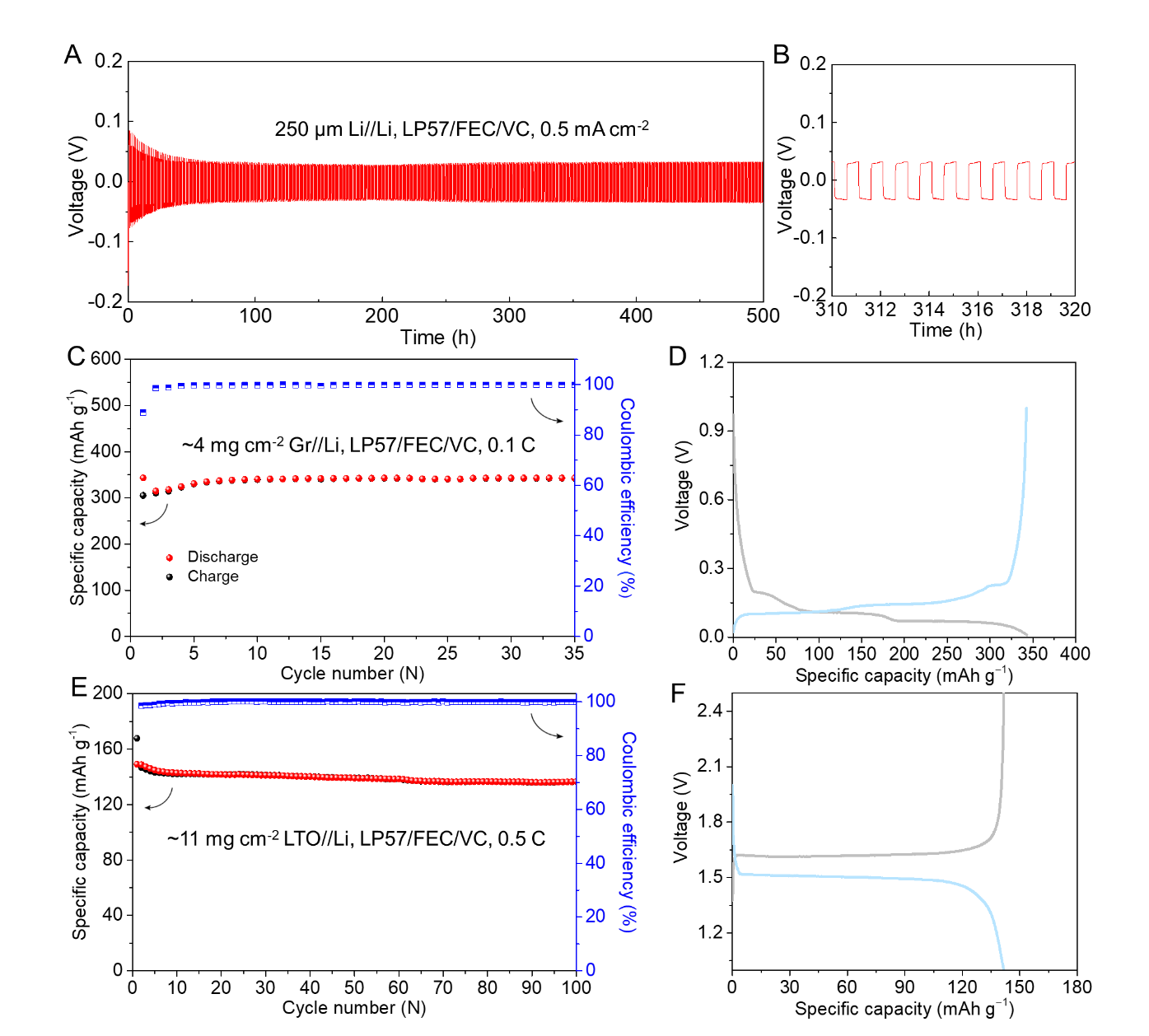


**Figure S13.** (A, B) Voltage profiles of Li//Li symmetrical cell using LP57/FEC/VC. (C) Cycling performance and (D) typical charge and discharge curve of Gr//Li cell with LP57/FEC/VC at 0.1 C. (E) Cycling performance and (F) typical charge and discharge curve of LTO//Li cell with LP57/FEC/VC at 0.5 C.


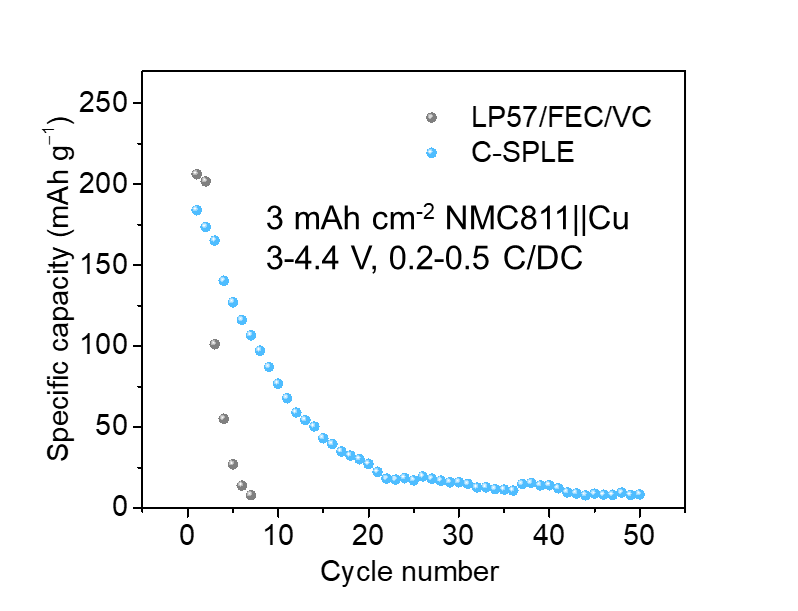


**Figure S14.** Cycling performance of NMC811||Cu batteries with C-SPLE and LP57/FEC/VC electrolytes.
